# Diets of infants and young children in two counties of Kenya: Key drivers and barriers to improvement

**DOI:** 10.1111/mcn.13334

**Published:** 2022-12-05

**Authors:** Judith Kimiywe, Hope Craig, Abigail Agyapong, Andrew Thorne‐Lyman, Patrick Matsisa, Laura Kiige, Patrick Codjia, Christiane Rudert, Stacy Katua, Rose Wambu, Betty Samburu, Penjani Kamudoni, Kudakwashe Chimanya, Stella Nordhagen

**Affiliations:** ^1^ Department of Food, Nutrition and Dietetics Kenyatta University School of Public Health and Applied Human Sciences Nairobi Kenya; ^2^ Department of International Health Center for Human Nutrition, Johns Hopkins Bloomberg School of Public Health Baltimore Maryland USA; ^3^ College of Agriculture and Life Sciences Cornell University Ithaca New York USA; ^4^ Department of International Health Center for a Livable Future, Johns Hopkins Bloomberg School of Public Health Baltimore Maryland USA; ^5^ Department of Mathematics and Statistics Jomo Kenyatta University of Agriculture and Technology Nairobi Kenya; ^6^ UNICEF Kenya Country Office Nairobi Kenya; ^7^ UNICEF Eastern and Southern Africa Regional Office Nairobi Kenya; ^8^ Kenya Ministry of Health Nairobi Kenya; ^9^ Global Alliance for Improved Nutrition (GAIN) Geneva Switzerland

**Keywords:** behaviour, beliefs, community, complementary feeding, food intake, infant and child nutrition

## Abstract

Infant and young child feeding (IYCF) practices are influenced by many context‐specific factors related to local food systems as well as social and cultural practices. Understanding these local contextual perspectives is essential for designing effective programs and policies. This paper uses focused ethnographic study methods to examine challenges experienced by mothers related to IYCF in two counties in Kenya, a country with considerable heterogeneity in agriculture, food systems, and cultures. A two‐phase qualitative study was undertaken in each of Kilifi County and West Pokot County, entailing interviews and rating activities with mothers, health workers, and vendors. Interviews were audio‐recorded, transcribed, translated into English, coded, and analysed by topic. Results show low levels of dietary diversity in both counties; in West Pokot, the level of adequate meal frequency is also low. Core foods in young child diets included maize porridge and family foods such as ugali (stiff maize porridge), vegetables, beans, fish, and plantains. Food safety, acceptability, and acquisition ease were the main drivers of food choice. Mothers generally felt that all core foods fed to young children are healthy and safe, but there was more variability regarding child acceptance, acquisition ease, cost, and convenience. Common barriers to feeding nutrient‐dense foods to children included child illness, economic constraints, and limited knowledge of modification strategies, skills, or tools to make the foods suitable for young children. Potential actions to address these barriers include sharing information on child‐appropriate recipes; raising awareness on local, affordable nutrient‐dense foods; and improving WASH practices to reduce illness frequency.

## Key points

- Children experience low levels of dietary diversity in Kenya's Kilifi and West Pokot counties; in West Pokot country, low meal frequency is also an issue.
- Core foods in young child diets included maize porridge and family foods like ugali, vegetables, beans, fish, and plantains.
- Food safety, acceptability, and ease of acquisition were the main drivers of food choice.
- Barriers to giving nutrient‐dense foods were illness, economic constraints, and mothers’ knowledge of food modification strategies.
- Sharing child‐appropriate recipes and information on affordable nutrient‐dense foods could help lower barriers.

## Graphical abstract

This rating of caregivers' impressions of dishes and food items for young children in Kilifi and West Pokot counties indicates that most foods were rated well for healthiness and food safety. There is greater variation for acceptance and acquisition, and particularly for cost and convenience.

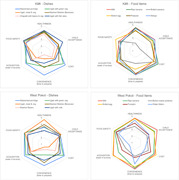

## 1 INTRODUCTION

The foods children are fed (including breastmilk) play an essential role in their nutrition, health, and development. For many years, encouraging exclusive breastfeeding for the first 6 months of life has been a major focus of nutrition‐ and infant mortality reduction‐related policies and interventions, as recommended by international bodies including the World Health Organisation (WHO) and UNICEF (Victora et al., 2016). Growing attention is now being paid to how food systems shape the diets of young children (6–24 months of age) (UNICEF, 2019, 2020). During this life stage, children transition from breastfeeding to solid foods, the eruption of teeth expands the foods they can consume, and they have growing autonomy to feed themselves (UNICEF & GAIN, 2019). Adequate diversity of foods is important for attaining nutrient adequacy in this age group and is associated with better child development outcomes (Bai et al., 2020; Muthini et al., 2020; Ruel et al., 2018), including brain development (Prado & Dewey, 2014). However, young children in Kenya often fail to achieve this, with only 38.3% of children 6–24 months meeting the minimum dietary diversity threshold (MDD, 4+ food groups of 7) (Kenya National Bureau of Statistics, Ministry of Health/Kenya, National AIDS Control Council/Kenya, Kenya Medical Research Institute, & National Council for Population and Development/Kenya, 2015).

Throughout East Africa and globally, the availability of foods in markets, food prices, seasonality, and purchasing power are important determinants of household food access (Bai et al., 2020; Ruel et al., 2018). Cultural and social norms around infant and young child feeding (IYCF) also play an important role in shaping which foods are fed to children and how. This includes whether or not responsive feeding techniques (which can help foster improved child growth and development (Hromi‐Fiedler et al., 2020; Vazir et al., 2013)) are used, the timing of introduction of different foods, and the frequency of meals/snacks. Other factors such as caretaker time availability, women's empowerment, and knowledge can also influence IYCF, but relationships vary by context (Kassie et al., 2020; Komatsu et al., 2018). Understanding local drivers of IYCF decisions is therefore important. Different approaches have been used to understand cultural dynamics around IYCF. One approach is focused ethnographic study (FES), which uses qualitative and quantitative research methods to understand the views of community members and interpret them within the context of nutrition science to inform programs (De Ver Dye et al., 2015; Dickerson et al., 2008; Pelto et al., 2013; Thuita et al., 2019).

Kenya is a country with significant heterogeneity in food systems and IYCF practices. The prevalence of MDD among children 6–24 months ranges from 14.7% in North‐Eastern Region to 67.9% in Nyanza (Kenya National Bureau of Statistics, Ministry of Health/Kenya, National AIDS Control Council/Kenya, Kenya Medical Research Institute, & National Council for Population and Development/Kenya, 2015). Such variation by context reinforces the importance of studying how local populations approach IYCF. Prior work using FES to explore IYCF in five counties (Thuita et al., 2019) found support for the idea that a cultural core exists around appropriate foods for young children across regions but also that variation exists across sites. A key gap remaining was exploring whether such findings applied to other parts of the country; this need was particularly acute for those counties with high levels of childhood malnutrition, such as Kilifi and West Pokot (Kenya National Bureau of Statistics, Ministry of Health/Kenya, National AIDS Control Council/Kenya, Kenya Medical Research Institute, & National Council for Population and Development/Kenya, 2015).

The objectives of the present research were to use adapted FES methods to examine young children's diets and the barriers and challenges experienced by caretakers in providing them with nutritious foods in two distinct counties in rural Kenya: West Pokot, an inland, dry, livestock‐focused area, and Kilifi, a coastal, humid, more agrarian area, to better understand how interventions could help improve practices within the 6–24‐month age group.

## 2 METHODS

### 2.1 Study location

Kilifi County is in coastal western Kenya with a hot, humid climate; part of the county has high rainfall and practices mixed agriculture (growing staples such as maize and cassava as well as vegetables, fruit, and nuts, for subsistence and cash), while livestock is the main activity in arid areas and fishing is common in coastal villages. West Pokot, in inland eastern Kenya, similarly has a moister area with mixed agricultural and agropastoral practices (growing staples such as maize and potatoes as well as legumes, vegetables, and fruit) and an arid area with pastoralist livestock‐keeping. West Pokot is less densely populated, and households have more limited market access. Both counties suffer from high levels of malnutrition, with 39% of children under five in Kilifi and 45% in West Pokot stunted, compared to the national level of 26% (Kenya National Bureau of Statistics, Ministry of Health/Kenya, National AIDS Control Council/Kenya, Kenya Medical Research Institute, & National Council for Population and Development/Kenya, 2015). The two counties were chosen due to limited prior study and local stakeholder priorities.

### 2.2 Respondent selection

FES uses two phases: in Phase 1, a small sample of key informants are interviewed about the main study topics; in Phase 2, a larger number of interviews delve in depth on topics emerging from Phase 1 (Pelto et al., 2013). FES is based on small samples, with representativeness assessed first through careful sampling and then through “saturation,” the situation in which no new insights are obtained with further interviews, typically achieved with 30–35 respondents (Pelto et al., 2013). Here, we used an adapted approach of the FES, focusing on primary caregivers but also including community health workers to obtain a perspective on general community‐level drivers and food vendors to understand market food availability.

Based on this, 8 and 32 caregivers were interviewed in Phases 1 and 2, respectively. To recruit them, first, random sampling was used to identify two sub‐counties in Kilifi and four in West Pokot as well as a set of health centres within each sub‐county. Within these, a rapid census of study communities was undertaken to list all households with children 6–24 months and their basic socioeconomic information. This list was divided into four child age groups (6–8, 9–12, 13–18, and 19–24 months). Respondents were then selected randomly from the list, selecting an equal number of respondents representing each child age group, as well as ensuring both younger (<25 years) and older mothers (>25 years) were included. While in principle all primary caregivers of young children were eligible for the study, in practice, due to local cultural norms, all those included were mothers (we, thus, refer henceforth to the interviewed caregivers as “mothers”). Community health volunteers (CHVs, *n* = 20 per country) and food vendors (*n* = 8 per county) were also interviewed in Phase 1 of each study. All CHVs who had been doing community health outreach in the target area for at least a year and were local residents were eligible, and selection was purposive, considering age and gender balance. Food vendors were chosen to cover established stores, temporary kiosks, and street food vendors, and a range of locations (i.e., central towns, remote areas).

### 2.3 Data collection and analysis

**Figure 1:**
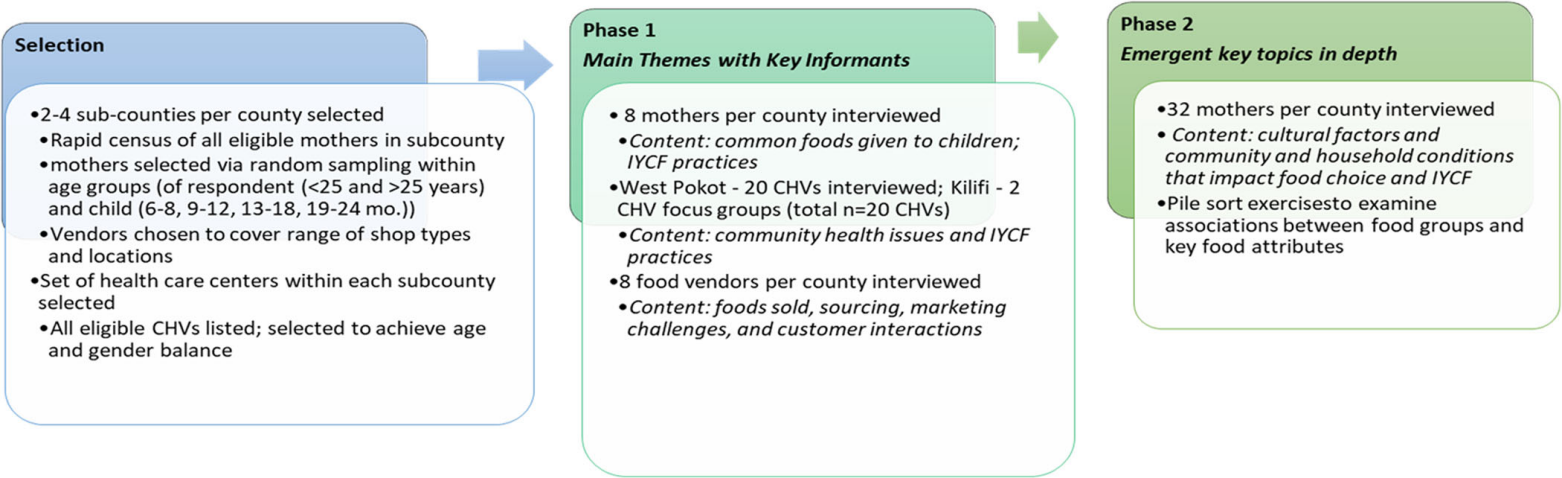
Summary of research phases, including respondents and topics covered. CHVs, community health volunteers; IYCF, infant and young child feeding

Data were collected in February and March 2020 in Kilifi and November 2020 in West Pokot. Methods employed included focus group discussions (FGDs) and semistructured interviews, which included food listings, pile sorting (using picture cards based on foods listed in Phase 1), and (for the CHVs) seasonal calendar activities (Pelto et al., 2013). Figure 1 summarises the number of interviews and the topics covered in each phase. Of note, while FGDs were conducted with CHVs in Kilifi, in West Pokot these topics were covered through individual interviews with CHVs instead, due to COVID‐19 protocols.

All interviews used a semistructured guide, which were based on those of Pelto and Armar‐Klemesu (2014), which have been extensively tested and used in several other regions of Kenya. The validated Pelto and Armar‐Klemesu guides were adapted slightly to include additional questions on food vendors (to reflect growing reliance on food purchase) and, in West Pokot, to include questions on Covid‐19 taken from a tested UNICEF module. Interview guides were further tested and refined through piloting in each county. Interviews were conducted in Kiswahili with translation into local languages, as needed. At least one member of each interviewing team was from the local community. Interviews were audio‐recorded, then transcribed and translated into English for analysis. Quantitative and short‐answer questions (e.g., demographic data, pile‐sort data) were entered via tablets to a secure Cloud‐based storage platform using Open Data Kit software. Data collection was closely supervised and reviewed to ensure high quality. Transcripts were coded and analysed using an approach that integrated grounded theory (generating themes to develop codes) with the start‐list method (relying on existing themes from the interview guides to develop codes); this approach allowed us to both derive themes from participant experiences and build on existing IYCF literature. Qualitative analysis used ATLAS.ti (ATLAS.ti Scientific Software Development GmbH), while the quantitative data were analysed using SPSS v.20 (IBM Corp).

### 2.4 Ethical considerations

Institutional Review Board approval was obtained from Kenyatta University (PKU/2018/I1166) and John Hopkins University (9825/MOD569). Permission was received from Kenya's National Council for Science and Technology and Innovation (NACOSTI/P/19/2475) and the Ministry of Health, Kilifi, and West Pokot Counties. Approval was also obtained from local administrators. In West Pokot, COVID‐19 prevention guidelines provided by the WHO, Kenya Ministry of Health, and West Pokot County were observed under the supervision of County and Sub‐County Ministry of Health response teams; this included social distancing, masking, outdoor interviewing where feasible, and hand sanitiser. All participants provided informed consent.

## 3 RESULTS

### 3.1 Respondent characteristics

Characteristics of mothers’ responses are shown in Table 1 and are broadly similar across counties. The typical mother was a woman in her 20s with primary education. Most respondents had 2–3 children, one of which was under age 5. However, it was not uncommon to have four or more children, or more than one child under age 5. Households in West Pokot were somewhat better off than those in Kilifi, but most households in each county earned less than 20,000 KSh a month (184 USD). Only one mother in Kilifi was in formal employment, whereas six (19%) in West Pokot were. Nearly all households had access to land and owned some livestock, particularly in West Pokot. While mobile phone access was widespread, few households had improved latrines (i.e., flush toilets or ventilated pit latrines) or used improved cooking fuels (i.e., gas or electricity as opposed to charcoal or wood).

**Table 1:** Caregiver respondent characteristics

	Kilifi		West Pokot	
	Number	%	Number	%
Respondent characteristics				
Age				
<20	6	18.8	3	9.4
20–29	18	56.3	24	75.0
30–39	4	12.5	12	37.5
>40	4	12.5	1	3.1
No education	4	12.5	3	9.4
Completed primary education only	19	59.4	10	31.3
Completed secondary education	3	9.4	11	34.4
Household characteristics				
Size, members				
3–5	9	28.1	14	43.8
6–10	16	50.0	14	43.8
11–15	5	15.6	3	9.4
>15	2	6.3	1	3.1
Household income, KSh^a^				
<8000	7	21.9	8	25.0
8001–20,000	11	34.4	13	40.6
20,001–40,000	10	31.3	5	15.6
>40,000	2	6.3	6	18.8
Has access to land	28	87.5	30	93.8
Owns chickens	25	78.1	30	93.8
Owns cattle	10	31.3	21	65.6
Owns goats	15	46.9	15	46.9
Has electricity^b^	7	21.9	8	25.0
Improved latrine^c^	4	12.5	4	12.5
Improved cooking fuel^d^	1	3.1	1	3.1
Owns mobile phone	30	93.8	29	90.6
Owns radio	13	40.6	24	75.0
Owns motorcycle	6	18.8	8	25.0
*n*	32		32	

*Note*: Includes data from the Phase 2 interviews only.

^a^ At the time of the research, 1 USD = approximately 105 KSh.

^b^ Electricity access was assumed based on the household's main source of light.

^c^ Improved toilets include flush and ventilated improved pit latrines not shared with other households.

^d^ Improved fuel includes gas or electricity.

In both counties, mothers were reported as being primarily responsible for IYCF. However, other family members (namely mothers‐in‐law and older children) also played a role in cooking, particularly when the mother was away. The child's father, maternal grandmother, and especially paternal grandmother influenced what the child was fed in other ways. The influence of the father was mainly on decision‐making, particularly purchasing food items for the child but also opining on what the child should be fed; for some mothers in West Pokot, however, the influence of the child's father was reported to be minor due to his being rarely home. The child's maternal grandmother and paternal grandmother (i.e., the mother's mother‐in‐law) were commonly named as having the most influence in IYCF: their opinion on topics such as childcare, breastfeeding, and timing and food choice for complementary feeding is respected due to their personal experience with raising children. These relatives’ role was larger in Kilifi, where households tended to consist of an extended (as opposed to nuclear) family, all living together. Health workers’ role was comparatively small and focused on education. A detailed exploration of the dynamics of these roles and how they influence decision‐making on IYCF is beyond the scope of this study but an important topic for future research.

### 3.2 IYCF practices

Key IYCF practices, defined according to (WHO & UNICEF, 2021), are shown in Table 2. While most young children were breastfeeding, the rate was significantly lower in the oldest age group. Minimum meal frequency was met by most children in Kilifi, but many of these meals consisted of watery porridge and black tea, with only 1–2 solid meals on normal days. In West Pokot, 41% of children met the minimum meal frequency. Less than 20% of children in either county met MDD (5+ of 8 food groups). Grains, roots, and tubers were consumed by nearly all children in both counties (91% in West Pokot, 100% in Kilifi), vitamin‐A‐rich fruits and vegetables were consumed by 41% in Kilifi and 56% in West Pokot, and dairy was consumed by all children in West Pokot but only 31% in Kilifi. Furthermore, only a third or less of children had consumed flesh foods, legumes/nuts, other vegetables and fruits, and particularly eggs, which was consumed by only 2 of 64 children across the counties.

**Table 2:** IYCF practices

	Kilifi		West Pokot	
	Number	%	Number	%
Currently breastfeeding, mo	27	84	27	84
Age 6–8	8	100	8	100
Age 9–11	8	100	8	100
Age 12–17	8	100	7	88
Age 18–24	3	38	4	44
Minimum dietary diversity met	2	6	1	3
Minimum meal frequency met	28	88	13	41
*n*	32		32	

Abbreviation: IYCF, infant and young child feeding.

*Note*: Includes data from the Phase 2 interviews only. Achieving minimum dietary diversity entails consuming five of eight groups, including breastmilk, within the past 24 h.

One focus of FES is understanding the “cultural core foods” for young children—that is, those central to young children's diets within a particular culture—and whether they differ from “core foods” for a household overall (Thuita et al., 2019). Table 3 presents these as well as secondary foods (culturally important foods, somewhat less salient than “core foods”). Complementary foods in both counties consisted mostly of maize meal porridge, the consistency of which varied depending on the child's age, with many young children subsisting on liquid diets of porridge, milk, milk tea, and only 1–2 solid meals per day. Once a child reached 12 months, they would eat family meals like ugali (a stiff‐texture maize porridge), vegetables, beans, *omena* (small dried fish) and other fish, and plantains, softened with tea or soup/stew. Most meals eaten by young children were also eaten by the rest of the household, without a special meal served just for the child. The “core” foods were largely the same across the counties, except that milk was consumed in large amounts in West Pokot, due to greater livestock holdings. Both counties experience periods of scarcity during the dry season, but interestingly there was little change reported in young child diets during periods of greatest food insecurity or abundance.^1^ Instead, households relied on purchasing (sometimes on credit) foods from vendors to fill gaps in their own‐produced supply and receiving food donations or loans from neighbours or relatives.

**Table 3:** Core and secondary foods for young children

Food (*see note for explanations of local foods*)	Percentage of respondents mentioning as a food for children
Kilifi	
*Core foods*	
Porridge	30 (93.8%)
Ugali + omena/fish	10 (31.3%)
Tea + mahamri	6 (18.8%)
Ugali + green vegetables	7 (21.9%)
*Secondary foods*	
Rice	4 (12.5%)
Potatoes	4 (12.5%)
Milk	4 (12.5%)
Fruits	5 (15.6%)
West Pokot	
*Core foods*	
Ugali with soup/stew	30 (93.8%)
Fresh milk	20 (62.5%)
Maize meal porridge	18 (56.3%)
Ugali with fresh or fermented milk	6 (18.8%)
*Secondary foods*	
Milk tea	7 (21.9%)
Boiled Irish potatoes	6 (18.8%)
Rice with beans or bean soup	6 (18.8%)
Mashed potatoes or bananas	6 (18.8%)
Mashed pumpkin	4 (12.5%)
Eggs	3 (9.4%)
Chapatti and soup	3 (9.4%)
Fruit (oranges, bananas, avocado, mangoes, and pawpaw)	15 (46.9%)

*Note*: The local food names referenced are as follows: ugali—thick maize‐based porridge; omena—small dried fish; mahamri—fried bread; and chapatti—wheat‐based flatbread.

### 3.3 Drivers of choice on foods given to young children

Caregivers’ decisions about what foods to feed young children are partly shaped by their perceptions of those foods’ attributes—that is, whether the foods are seen as healthy, safe, affordable, easy to access, accepted by the child, and convenient for the caregiver (domains identified based on (Pelto & Armar‐Klemesu, 2014)). In a rating exercise with mothers, nearly all attributes were considered “very important,” confirming their relevance. Interviews also asked respondents how they conceptualised each of these attributes—that is, what things make food healthy, or what they consider “convenient” related to IYCF. Considering *healthiness*, mothers in both counties noted several aspects of what makes diets/foods “healthy”: a variety of foods; nutritious foods; enriching foods; observing good hygiene/cleanliness; and appropriate feeding frequency. There were few differences across the two counties regarding these attributes of “healthiness,” but there was some variation in what a “nutritious” food was seen to be. Mothers in Kilifi noted bananas, milk, oranges, and baby porridge, whereas those in West Pokot noted eggs, milk, fruits, and vegetables; specifically asked about which foods give strength, mothers in both counties noted ripe bananas, avocado, and enriched porridge. Some mothers specifically noted the presence of vitamins and protein in certain foods, like fruits and vegetables. Within “variety,” mothers noted both feeding diverse meals (e.g., porridge from multiple grains, legumes, milk) and feeding varied foods across time.

*[When deciding what to feed my child], I consider the health of the child. The weight of the child, for example. If the child weight is low, I change the food of the child. For example, I give eggs, pumpkin and beans. If the child has good weight, I continue with the usual diet of ugali, milk and porridge*. 28‐year‐old mother, West Pokot

Enrichment of foods included adding milk, margarine, and sugar or other flavourings (particularly to porridge); micronutrient powders or other vitamins were only mentioned within the context of the hospital and not commonly used at home. Meal frequency and timing entailed feeding the child snacks between meals and feeding food at the right time. Observing good hygiene including handwashing, cleaning utensils and the cooking/eating environment, and covering food when cooking and before storage. As one 24‐year‐old mother in West Pokot noted, “I consider the cleanliness of utensils.… So that baby does not get sick.” Thus, *food safety* was seen as being one aspect of healthiness.

Regarding *acceptability*, mothers named six core aspects that were shared across the counties: hunger; child health status; food consistency; food tastiness; encouragement and interaction during feeding; and child preferences. Hungry and healthy children were known to be more ready to eat, and foods that aligned to the child's specific preferences were more readily accepted. Regarding taste and consistency, mothers noted that adding ingredients like sugar or margarine increased food's acceptability for the child and that foods that were easier to swallow (e.g., porridge) were more readily eaten. In terms of encouragement during feeding, mothers noted that playing with the child, singing, talking softly, and sitting nearby helped increase the child's acceptance of food. As one 28‐year‐old Kilifi mother explained, “I think the relationship or the interaction the mother and child have affect how the baby feeds. This is because if baby is happy and playful, they will eat without fights.” In Kilifi, mothers also noted that familiar foods helped increase acceptability, with it being difficult to get a child to accept new food, and mentioned using glucose to stimulate a child's appetite; a West Pokot mother noted using multivitamins for the same purpose.

In terms of *convenience*, mothers’ responses were similar across the two counties and included ease of preparation; short cooking times; the amount of firewood needed to cook; and ease of acquisition. Acceptability was also noted as being related to convenience, as having food not be accepted by the child was time‐consuming and might lead the mother to force‐feed the child (to avoid the child being hungry or the food being wasted). Overall, it was clear that preparation time was the primary criterion for convenience; this favoured ready‐to‐eat foods like fried potatoes or quick‐prep foods like milk, whereas foods like beans and meat were noted as time‐consuming. Ease of acquisition (i.e., being available nearby) was also important, as it avoided time and transport costs; in Kilifi, some respondents noted a perceived need to buy what the local shopkeeper had in stock to maintain a good relationship and access credit in the future.

**Figure 2:**
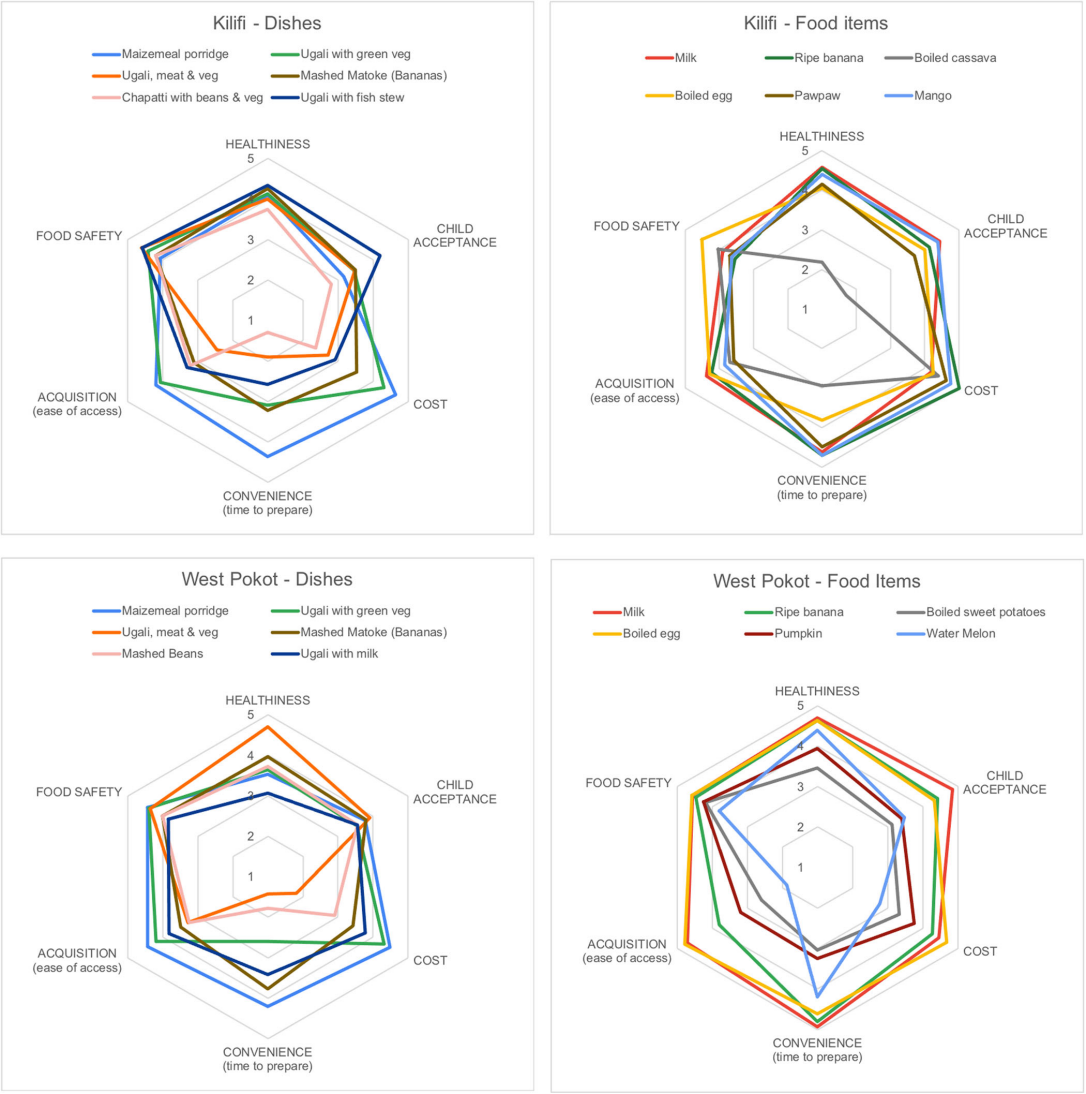
Rating of young child dishes and food items in Kilifi and West Pokot

Mothers were asked to rate the core infant and young child foods mentioned by respondents in the first study phase on each of these attributes, using a 5‐point scale with 5 being the most positive rating—then asked to explain the reasons behind their ratings. Figure 2 presents radar charts visualising these associations. Most foods were rated positively for healthiness and food safety; the only foods not seen as being generally healthy for young children were boiled cassava (in Kilifi) and ugali with milk (in West Pokot); no core foods for children were rated as much below a 4 (out of 5) on the “food safety” dimension. Mothers thus generally agree that all core foods fed to young children are healthy and safe. However, ratings were more variable for child acceptance and acquisition, and particularly for cost and convenience. Focusing on cost, dishes and foods noted as less affordable in West Pokot included ugali with meat and vegetables and mashed beans, watermelon, sweet potato, and pumpkin; in Kilifi, chapatti with beans and vegetables, ugali with meat and vegetables, and ugali with fish stew were less affordable dishes whereas all foods were considered fairly affordable. The less‐affordable dishes were also generally seen as less convenient to prepare and, to a lesser extent, harder to access. These dishes are all less‐commonly‐fed dishes for young children but are more nutrient‐dense than the alternatives (e.g., maizemeal porridge). Interestingly, both egg and milk were seen as relatively affordable, acceptable, convenient, safe, healthy, and easy to access in both counties, particularly West Pokot—but were rarely fed to children (particularly for egg). Maizemeal porridge (the core dish of young child diets) also rated well on all dimensions across both counties; the slightly lower score for acceptability was due to the belief that children would not accept it if not enriched with milk and sugar.

### 3.4 Barriers and challenges to improved IYCF

As shown in Section 3.1, key gaps in child diets in both counties were insufficient frequency of solid meals and, particularly, poor dietary diversity, with limited consumption of nutrient‐dense foods such as fish, meat, eggs, legumes/nuts, and, in Kilifi, dairy. In Section 3.2, mothers associated some of these foods (e.g., legumes, meat) with certain negative characteristics, particularly regarding convenience, but for others, the reason was less clear. The almost nonexistent feeding of eggs to children, despite widespread poultry keeping across both counties and strong ratings on all food attributes, was particularly striking. There were no reported myths associated with the consumption of eggs in Kilifi county, but in West Pokot, they were named by 28% of mothers as being “bad” for young children, due to a local traditional belief that it might lead to delayed speech. As one 22‐year‐old mother explained, “I have not given [eggs] because they say that the baby will not be able to speak early”—later clarifying that “they” were older women in the community. In both counties, the low consumption seemed driven primarily by economic motivation: eggs and chickens were sold for income (or kept to hatch more chickens). A secondary barrier was a lack of knowledge of modification strategies for safely feeding eggs and meat (and other nutrient‐dense foods such as fish) to young children: when asked why such foods were not fed, many mothers cited concerns about the child's lack of teeth and inability to chew.

Considering other nutrient‐dense foods, green leafy vegetables are traditionally used in both study areas as an accompaniment for ugali in family meals. However, children were reported to only eat the soup, not the whole vegetable. Beans and bean soups were served with secondary foods (e.g., rice, potatoes, chapati), but again children were reported to only eat the soup, not the whole beans, due to inability to chew (according to mothers)—further underlining the finding on lack of knowledge of modification strategies. As an 18‐year‐old mother in Kilifi noted, “[I don't give my child chapatis because] he cannot chew it; I even have not given him meat because he cannot chew. I only give him the soup.”

One nutrient‐dense food that was commonly given to children was omena, even though neither county harvested it. Omena (sometimes modified by grinding to flour) were considered to be healthy for children in Kilifi and, to a lesser extent, West Pokot and were thus purchased for them; though some mothers noted the cost as high, it was not seen as prohibitive.

A common challenge reported with IYCF in both counties was children's refusal to eat, which led to force‐feeding, medication (e.g., multivitamins, herbal remedies), or skipped/incomplete meals. Some felt this was due to the child being bewitched:

*Maybe the child ate yesterday very well and today he changes you may give milk or porridge and he refuses… Here, at our place, we believe that someone can look at your baby with "bad eye" and the child stops eating. [To treat this], you give animal meat with fat (like pig meat) or a drop of Murgus [a local herbs]… it will help relieve the problem, that is if someone passed‐ by with bad eyes on the baby*. 28‐year‐old mother, West Pokot

However, others felt it was due to illness. A concern emerging prominently in both counties was mothers’ fears of children being ill and, particularly, suffering from diarrhoea. Most mothers linked this to teething, which they reported led to fever, diarrhoea, and sometimes refusal to eat*.* Fear of diarrhoea was one reason for not feeding children, or not feeding them certain foods. For example, one 23‐year‐old mother in the Central Pokot sub‐county noted, “When the baby eats Sukuma wiki [local green], it gets diarrhoea, so I stopped feeding that vegetable.”

Indeed, when mothers in Kilifi were asked to list foods that were bad for young children, the main issues they mentioned had to do with either causing disease (diarrhoea, stomachache) or lack of knowledge of modification strategies to feed them safely, as opposed to “taboos” related to specific foods: foods mentioned in Kilifi included ugali with *mchunga* (rabbit grass) and family leftovers, both because the young child would be unable to chew the food well; leftovers were also cited as risking insect infestation, and black tea with chapatti or fried bread was named as not adding any nutrients to the child's diet. In West Pokot, mothers noted cold food (causing diarrhoea), boiled maize (unable to be chewed), *githeri* (a maize and bean dish; unable to be chewed and causes stomachaches), and beans (cause stomachaches) as well as sugary foods (causing later refusal of non‐sugary foods) and eggs (due to the abovementioned taboo).

Seasonal food shortages and a lack of money to buy food were also commonly named challenges in West Pokot, and a lack of time to feed (due to farm work, fetching water, and similar) was also named by a few respondents.

*During dry season, the cows migrate, and we are left without milk…. Getting water is also a problem … we get it from far distant places, so I have no time to be with the baby. Also, sometimes I might not have money to buy food, and also sometimes food could not be available in our market.* 30‐year‐old mother, West Pokot

## 4 DISCUSSION

This study of IYCF in two counties in Kenya revealed key gaps in child diets: insufficient frequency of solid meals and, particularly, poor dietary diversity, with limited consumption of nutrient‐dense foods. Three key barriers emerged as explaining these behaviours: a lack of knowledge of strategies for modifying family foods to be suitable for young children; economic constraints on accessing nutritious foods; and frequent child illness (leading to a fear of causing it by feeding the ‘wrong’ foods). In contrast, lack of knowledge of appropriate practices did not emerge as a key barrier: most mothers were able to articulate the importance of good nutrition, including concepts such as dietary diversity and meal frequency (Kimiywe & Chege, 2015).

Among these three barriers, the first is the most feasible to address through potential interventions. There are numerous known, culturally appropriate Kenyan recipes for creating meals from foods such as eggs, fish, and greens that can be easily eaten by young children—e.g., DiGirolamo et al. (2022) present an evidence‐based recipe book developed by a multidisciplinary consortium based on extensive formative research—and caregivers could be trained to prepare them. In so doing, it will be important to address their fears about children's inability to digest such foods and ensure the recipes are not overly time‐consuming, as that is a key motivator of mothers’ choices regarding foods for young children; interactive cooking classes and demonstrations could help dispel both fears.

The second barrier, economic realities of high prices compared to low incomes, is a challenging constraint that limits rural Kenyan households’ ability to access sufficient nutritious food, including for young children. It is estimated that 47.5% and 79.1% of Kenyans cannot afford a nutrient‐sufficient and healthy diet, respectively (FAO, IFAD, UNICEF, WFP, & WHO, 2020). However, recent research indicates that in the surrounding regions (Rift Valley for West Pokot and Coast for Kilifi), multiple foods exist that could provide a large share of nutrient requirements at an affordable price (the exception is iron in West Pokot, for which there are no affordable foods) (Ryckman et al., 2022). These include omena (for calcium, zinc, and vitamin B_12_), beans (for folate, plus iron in Coast province), liver (for vitamins A and B_12_, plus folate in Rift Valley), and carrots (for vitamin A). While omena emerged here as a widely acceptable complementary food, the others were not commonly named; there may be an opportunity to raise awareness on the nutritional value and affordability of these locally available foods. Where such affordable options are not available, cash transfers can be considered—the Nutrition Improvement for Children through Cash and Health Education project is currently testing this approach in Kilifi, West Pokot, and three other counties. In the long term, work can be done to reduce the cost of nutrient‐dense foods through interventions within supply chains (Traore et al., 2022) and to increase incomes. Both food systems and social protection systems thus play key roles in lowering this barrier.

In terms of child illness, mothers generally linked this to teething or intolerance of certain foods. While this is likely an accurate reflection of their perceptions, additional study results not reported here pointed towards other potential explanations. It is clear in the responses regarding food safety/hygiene that mothers had some knowledge of the importance of good hygiene when feeding children; however, this was not always practiced. Access to improved toilets was rare; access to safe drinking water in these areas is also imperfect (only 60% of rural Kenyan households have access to improved water sources (Kenya National Bureau of Statistics, Ministry of Health/Kenya, National AIDS Control Council/Kenya, Kenya Medical Research Institute, & National Council for Population and Development/Kenya, 2015). In both counties, this was a particular concern in dry season when some water sources are unreliable. In addition, most mothers were storing food for up to 12 h in a hot, humid, potentially unclean environment that could foster contamination; in West Pokot, 30% of mothers reported storing children's food for over 6 h. About two‐thirds of households in each county did not boil/treat drinking water; while most knew it was recommended, lack of time, fuel, or money prevented it. It thus seems likely that poor food safety practices are one contributor to children's frequent illness (DHS data confirm that about 25% of Kenyan children 6–24 months had diarrhoea in the two weeks before the survey (Kenya National Bureau of Statistics, Ministry of Health/Kenya, National AIDS Control Council/Kenya, Kenya Medical Research Institute, & National Council for Population and Development/Kenya, 2015). This can be addressed in the short term through training on optimal WASH practices coupled with counselling on feeding young children during illness and in the long term by improving access to infrastructure, particularly toilets and electricity to enable refrigeration. Both WASH and health systems thus play key roles in lowering this barrier.

Considering the study results within the context of prior FES work, they corroborate the results of others (Pelto & Thuita, 2016; Thuita et al., 2019) of there being a “cultural core” of IYCF foods across many regions of Kenya: porridge, milk, ugali, and tea all emerged as core foods both in that study and here; fish and leafy greens, however, were not part of that shared cultural core but were core in Kilifi (a coastal, horticultural region). As in that study, diets of young children also show considerable overlap with broader family diets; families’ eating habits have been shown to gradually transform a child's tastes and food preferences, ultimately teaching them to like what they eat (Kruger & Gericke, 2003). This underlines the importance of focusing nutrition interventions not only on young children but on the whole family, all of whom could likely benefit from improved diets; such interventions should also be inclusive of diverse members of the community, as food beliefs can vary by ethnic background (Mandelbaum et al., 2019). The present study's findings on eggs also add to the results of Pelto and Thuita (2016): in that study, egg taboos were found to exist in two of four counties (one also related to delayed speech), while eggs were seen as a core food in two others, underscoring diverse beliefs on their suitability for IYCF (Pelto & Thuita, 2016; Shitemi et al., 2018).

This study has several limitations. First, like most qualitative studies, it used a small, nonrandom sample, so results have uncertain generalisability. Several topics that influence IYCF practices—such as social norms (Dickin et al., 2021), gender roles (Martin et al., 2020), caregiver capabilities (Matare et al., 2021; Oteri et al., 2020), seasonality, market availability, and land access—were covered in the broader research but not here due to space limitations. Data from West Pokot were collected during COVID‐19, which may have influenced results (see Box 1). Finally, the study was focused only on primary caregivers. In these communities, these were all mothers, but other household and community members also play an important role in influencing young child feeding and it is important for IYCF interventions to engage with them, also (Bar‐Yam & Darby, 1997; Kuyper & Dewey, 2012; Mukuria et al., 2016; Muraya et al., 2017). In these communities, grandmothers/mothers‐in‐law played a particularly important role in influencing IYCF practices, and fathers played a role in purchasing foods for children. A study that interviewed fathers may, for example, have yielded different implications related to economic constraints on accessing nutritious foods, whereas one interviewing mothers‐in‐law may have helped add nuance to the analysis of knowledge gaps related to food modification strategies. Future studies should broaden their samples to capture the views of these additional caregivers and influencers as well as other aspects of the social organisation of nutrition (Tumilowicz & Pelto, 2020).

Box 1 **Impact of Covid‐19 on child feeding practices in West Pokot**

As the study in West Pokot happened during the COVID‐19 pandemic, while that in Kilifi happened just before, the potential impact of the pandemic on results must be considered. Indeed, livelihoods in West Pokot were greatly affected by the pandemic: households lost jobs and businesses they relied on for income, and restrictions of movement forced farmers to sell their produce locally, at very low prices. In addition, some families reported increased food and transportation prices and difficulties accessing a wide variety of foods, likely having a negative impact on IYCF. Children were reported to eat on average two meals a day, as opposed to three before COVID, and some mothers were concerned that their children were losing weight because of poor feeding. In addition, the fear of contracting COVID was reported by mothers as a reason not to go to health facilities for services such as growth monitoring and counselling, as well as to treat a sick child, perhaps further exacerbating the situation. On a positive note, mothers noted that, since they were now home all the time, they had more time with their children

*Many parents are ensuring that children do not go to their neighbours so that they do not get COVID from them. Before then, children could go to their neighbours and feed from there.* CHV, West Pokot

*It affects because we have to stay home and we cannot go out to earn an income… l can't sell vegetables, l have to stay at home… Expenses have gone up, since food prices have increased*. 34‐year‐old mother, West Pokot

The results must also be interpreted in light of the pandemic. While there are no obvious ways in which the influence of the pandemic has led to differences in main results across the two counties, we cannot say for sure, and there are somewhere such differences could be envisioned. For example, the greater importance mothers in West Pokot gave to food safety as a motivator of food choice could be linked to greater attention to health concerns; lower ratings for ease of acquisition for certain foods could be due to COVID‐related supply chain disruptions.

At the same time, this in‐depth, focused research in two counties of rural Kenya has provided an in‐depth examination of mothers’ perspectives, indicating context‐specific drivers of suboptimal IYCF practices. These insights strengthen the evidence base for designing potential interventions across food, health, WASH, and social protection systems.

## CONFLICT OF INTEREST STATEMENT

The authors declare no conflict of interests.

## AUTHOR CONTRIBUTIONS

Judith Kimiywe, Hope Craig, and Andrew T. Lyman designed the research. Judith Kimiywe performed the research with support from Hope Craig, Stacy Katua, and Patrick Matsisa. Judith Kimiywe, Patrick Matsisa, Hope Craig, and Abigail Agyapong analysed the data. Stella Nordhagen, Judith Kimiywe, Hope Craig, and Andrew T. Lyman wrote the paper. All authors contributed to writing and have read and approved the final manuscript.

## Data Availability

The data are not being made public at present due to ethical issues (namely. the difficulty with fully deidentifying qualitative data). Researchers interested in further use of the data may contact the authors to discuss the possibility of data sharing.

## Footnotes

^1^
The dry season in Kilifi is in January–March and to a lesser extent July and August, data were collected in February; in West Pokot, the dry season is December–March, and data were collected in October and November.
